# Nuclear Lamins and Emerin Are Differentially Expressed in Osteosarcoma Cells and Scale with Tumor Aggressiveness

**DOI:** 10.3390/cancers12020443

**Published:** 2020-02-13

**Authors:** Enrica Urciuoli, Stefania Petrini, Valentina D’Oria, Martina Leopizzi, Carlo Della Rocca, Barbara Peruzzi

**Affiliations:** 1 Multifactorial Disease and Complex Phenotype Area, Research Center, Bambino Gesù Children’s Hospital, 00165 Rome, Italy; enrica.urciuoli@gmail.com; 2 Confocal Microscopy Core Facility, Research Center, Bambino Gesù Children’s Hospital, 00165 Rome, Italy; stefania.petrini@opbg.net (S.P.); valentina.doria@opbg.net (V.D.); 3 Department of Medico-Surgical Sciences and Biotechnology, Polo Pontino, Sapienza University, 04100 Latina, Italy; m.leopizzi@hotmail.it (M.L.); carlo.dellarocca@uniroma1.it (C.D.R.)

**Keywords:** osteosarcoma, lamins, nuclear envelope, confocal microscopy, tissue microarray

## Abstract

The nuclear lamina is essential for the maintenance of nuclear shape and mechanics. Mutations in lamin genes have been identified in a heterogeneous spectrum of human diseases known as “laminopathies” associated with nuclear envelope defects and deregulation of cellular functions. Interestingly, osteosarcoma is the only neoplasm described in the literature in association with laminopathies. This study aims characterized the expression of A-type and B-type lamins and emerin in osteosarcoma, revealing a higher percentage of dysmorphic nuclei in osteosarcoma cells in comparison to normal osteoblasts and all the hallmarks of laminopathic features. Both lamins and emerin were differentially expressed in osteosarcoma cell lines in comparison to normal osteoblasts and correlated with tumor aggressiveness. We analysed lamin A/C expression in a tissue-microarray including osteosarcoma samples with different prognosis, finding a positive correlation between lamin A/C expression and the overall survival of osteosarcoma patients. An inefficient MKL1 nuclear shuttling and actin depolymerization, as well as a reduced expression of pRb and a decreased YAP nuclear content were observed in A-type lamin deficient 143B cells. In conclusion, we described for the first time laminopathic nuclear phenotypes in osteosarcoma cells, providing evidence for an altered lamins and emerin expression and a deregulated nucleoskeleton architecture of this tumor.

## 1. Introduction

The nuclear lamins are type V intermediate filament proteins that polymerize to form a highly-organized meshwork between the inner nuclear membrane (INM) and the chromatin. They are essential for the maintenance of nuclear shape and mechanics, gene regulation, chromatin organization, chromosome positioning, DNA replication and repair, and signaling regulation [1,2]. Lamins are grouped into A-type and B-type depending on their structural and biochemical properties, and their expression is regulated during development in a cell-type-specific manner. In mammals, the *LMNA* gene gives rise to lamins A and C, but also minor isoforms as lamin C2 and Adelta10, by alternative RNA splicing [3], whereas B-type lamins are encoded by the *LMNB1* (lamins B1) and *LMNB2* (lamins B2 and B3) genes [4,5,6]. B-type lamins are ubiquitously expressed and considered essential for cell survival, whereas A-type lamins change during development and cell differentiation stages, being absent in embryonic stem cells as well as in induced pluripotent stem cells (iPSCs) [7,8,9]. A-type lamins bind to B-type lamins and to several structural proteins, including the integral INM protein emerin, nesprins, lamina-associated polypeptide 2 isoform α (LAP2α), NUP153, SUN-domain-containing proteins, and nuclear actin thus forming a structural network essential for nuclear integrity and nucleo-cytoskeletal coupling [10,11]. Both A- and B-type lamins are localized in the nucleus lamina, and A-type lamins are also expressed in the rest of the nucleoplasm as they are non-farnesylated proteins after maturation steps [12,13].

*LMNA* mutations have been identified in a heterogeneous spectrum of rare human diseases commonly known as “laminopathies” [3,14,15] involving different tissues and multiple systems with features of accelerated aging. The most severe laminopathies are progeroid syndromes including the premature aging disease Hutchinson-Gilford progeria syndrome (HGPS), “atypical” Werner’s syndrome (WS), restrictive dermopathy and mandibular acral dysplasia. In particular, HGPS is caused by a *LMNA* point mutation responsible for an aberrant and truncated prelamin A called progerin (laminA Δ50), that tightly associates with the INM and accumulates intranuclearly, damaging nuclear architecture and cellular function [16].

Conversely, defects in B-type lamins are rare events and reported in some genetic diseases as the adult-onset autosomal dominant leukodystrophy (ADLD) associated to *LMNB1* duplication or *LMNB1* promoter mutation [17,18], and the partial lypodystrophy associated to *LMNB2* heterozygous mutations [19,20,21].

Alterations in the expression of A- and B-type lamins and nuclear lamina-associated proteins have recently been explored in cancer development, tumor propagation and progression, and several reports have suggested their involvement in prostate cancer, hepatocarcinoma, breast and lung cancer [12,22,23,24,25]. Interestingly, although cancer development in laminopathic patients is a rare event, osteosarcoma is the only neoplasm associated to cases of HGPS and WS syndromes [26,27,28,29]. Osteosarcoma, the most common primary malignant bone tumor in children and adolescents [30,31], is a highly aggressive cancer that metastasizes primarily to the lung [32,33]. Osteosarcoma arises from cells of the mesenchymal origin and is characterized by the production of malignant osteoid by pleomorphic malignant cells within the connective tissue matrix [34].

Although nuclear lamins have key pivotal roles in driving the differentiation of mesenchymal stromal cells towards osteogenic lineage [35,36], the composition of nuclear lamina proteins has been not investigated in osteosarcoma cancer cells. In this paper we compared the nuclear phenotype of osteosarcoma cells with increasing aggressiveness [37,38] to normal osteoblasts and deepened the relationship between expression changes of pivotal nuclear envelope (NE) components, as A- and B-type lamins and emerin, in osteoblasts and osteosarcoma cell lines and their potential malignancy by confocal microscopy, biochemical and RT-PCR analyses. The behavior of these NE components was analyzed in bone tissue sections from controls and patients affected by high and low grade of osteosarcoma in order to assess a correlation between lamins and emerin immunoexpression and their prognostic relevance. Further, the functional effects of A-type lamin alterations was investigated by the analysis of immunoexpression and subcellular distribution of (MKL1), protein Retinoblastoma (pRb) and Yes-Associated Protein (YAP), known as functional sensors of A-type lamin perturbations, in osteoblasts and osteosarcoma cell lines.

MKL1, a mechanosensitive transcription factor belonging to the family of Myocardin-related transcription factors (MRTFs), is a coactivator that, following serum stimulation, translocates into the nucleus and physically associates with the Serum Response Factor, thereby transducing cytoskeletal signals to the nucleus and activating a subset of serum response factor-dependent genes that promote differentiation and cytoskeletal organization [39]. Lamin-A/C-deficient (*Lmna*^−/−^) mice have impaired nuclear translocation and downstream signalling of MKL1, due to an altered actin dynamics [40]. More in details, the work of Willer and coauthors demonstrated that emerin stimulates SRF−MKL1-dependent gene activity in a substrate stiffness-dependent manner. Specifically, emerin was required for MKL1 nuclear accumulation and maximal SRF−MKL1-dependent gene expression in response to serum stimulation of cells grown on stiff substrates but was dispensable on more compliant substrates [41].

The Retinoblastoma protein (pRB), an oncosuppressor and a key regulator of cell fate (proliferation/differentiation), is another factor interacting with A-type lamin. These physiological interactions are hampered in laminopathies characterized by muscle dystrophy [42] and *Lmna^−/−^* mouse fibroblasts, showing reduced levels of pRb, exhibited cell cycle defects [43]. In the context of mechanosensing genes related to lamin A/C, we also assessed the subcellular localization of the Yes-Associated Protein (YAP), which has been described to correlate with the stiffness of the extracellular matrix and to be dependent on lamin A/C function [36,44].

## 2. Results

### 2.1. Osteosarcoma Cells Exhibit Laminopathic Nuclear Phenotypes

**Figure 1:**
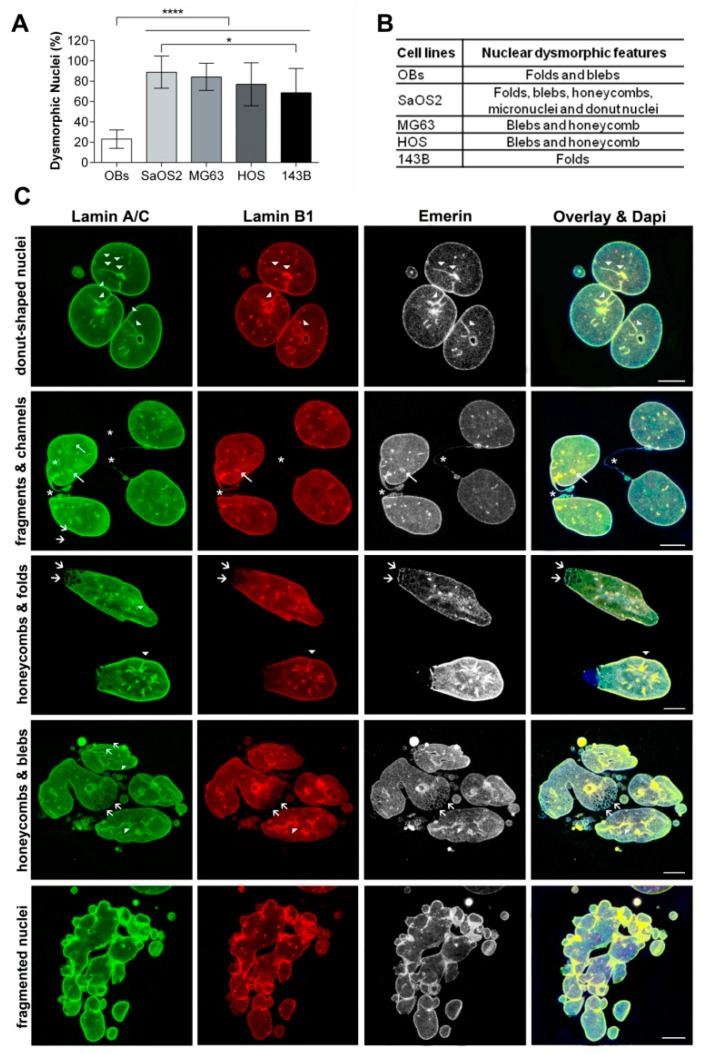
(**A**) Relative percentage of abnormal nuclei observed in osteoblasts (OBs) and osteosarcoma (SaOS2, MG63, HOS and 143B) cell lines. * *p* < 0.05; **** *p* < 0.001. (**B**) Morphological features of nuclear dysmorphisms such as folds, honeycombs, fragmented, lobulated (blebs) and donut nuclei, are differentially observed in each cell line. (**C**) Morphological aspects of SaOS2’s nuclear envelope/*lamina* exhibiting dysmorphic features resembling those of laminopathic nuclei highlighted by multiple labeling with lamin A/C (green), lamin B1 (red) and emerin (white) antibodies. Donut-shaped nuclei (first lane), nuclei with folds (arrowheads, first and third lanes) and channels (second lane, arrow) micronuclei and fragmented NEs (second lane, asterisk; fourth and fifth lanes) are shown. Further lamin A/C and emerin meshworks lacking lamin B1 (honeycombs, third and fourth lanes, arrows) are also observed. Nuclei were stained with Dapi. Bars: 10 μm.

By evaluating the frequency of morphological nuclear changes in comparison to normal osteoblasts, osteosarcoma cell lines showed a higher percentage of dysmorphic nuclei (*p* < 0.001 by one-way ANOVA analysis; Kruskal–Wallis test) (Figure 1A,B), resembling most of the phenotypes observed in laminopathic nuclei, as folds, lobulations, honeycombs, donut nuclei, and micronuclei (Figure 1C).

Interestingly, SaOS2, commonly recognized as low aggressive osteoblast-like cells, showed the highest percentage of nuclei with irregular outlines and the presence of all the hallmarks of laminopathic nuclei. In contrast, 143B cells, the most aggressive osteosarcoma cell line, showed the lower percentage of nuclear deformations among osteosarcoma cells, consisting in folded and lobulated nuclei (SaOS2 vs. 143B: *p* < 0.05 by one-way ANOVA analysis; Dunn’s multiple comparison test). This result prompted us to deeper investigate nucleoskeleton architectures and NE protein expression in osteoblasts and osteosarcoma cells.

### 2.2. Differential Expression of A/C and B1 Lamins and Emerin in Osteosarcoma Cell Lines Correlates with Their Nucleoskeleton Architectures

The expression and distribution of lamin A/C, lamin B1 and emerin were investigated by confocal laser scanning microscopy, western blot analysis and RT-PCR, and osteoblasts were considered as reference cells for the osteosarcoma cell lines.

#### 2.2.1. Osteoblasts (OBs)

**Figure 2:**
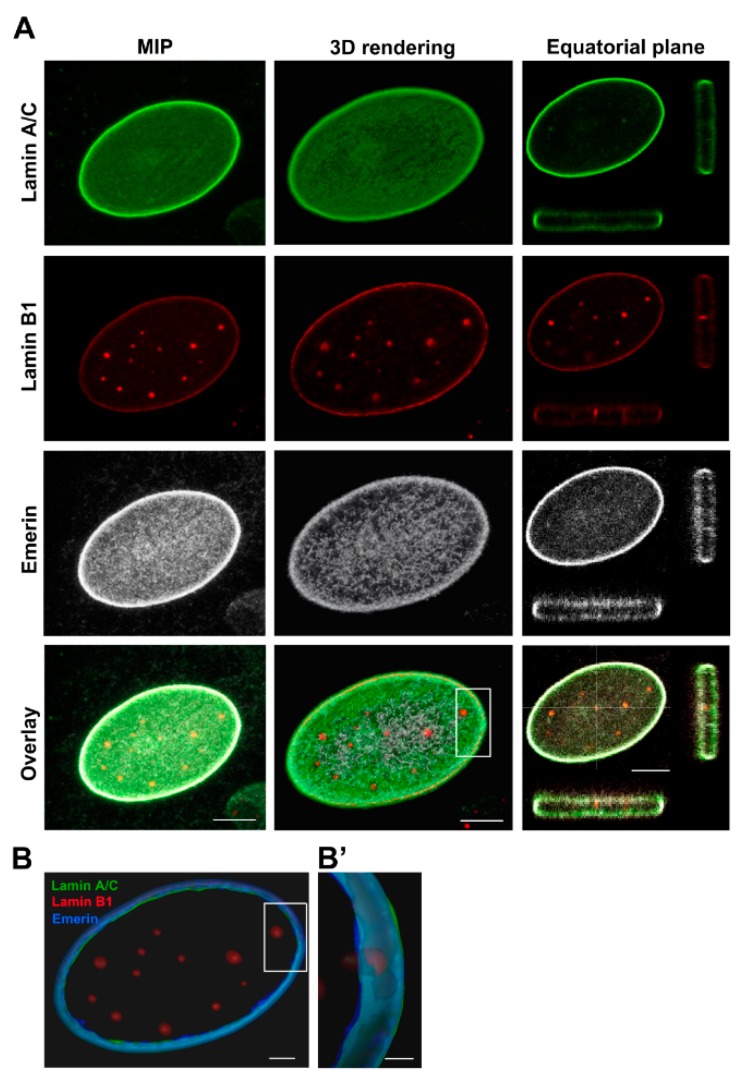
Confocal imaging of co-distribution of A/C and B1 lamins and emerin in osteoblasts. (**A**) Maximum intensity projection (MIP) of series of z-stacks images covering the entire nucleus (left column), 3D rendering in blend volume mode (central column, LAS X 3D software, Leica Microsystems, Wetzlar, Germany), and XZ- and YZ-axes projections performed in the central focal plane of the nucleus (right column) are shown. Bars: 5 µm. (**B**) 3D rendering in the focal central plans of the nuclear rim in surface volume mode (Imaris software). (**B’**) High magnification of the 3D rendering of the boxed area showing the differential expression of lamins (in green and red) and emerin (pseudocoloured in blue). Bars: 1 µm.

In OBs we detected irregular morphologies in about 23.07 ± 9.02% of nuclei showing blebs and folds. In most of the cells assessed, the nuclear rim was regularly shaped (Figure 2A), and showed a brilliant emerin expression, an intense staining for lamin A/C and a low expression of lamin B1—the latter was also concentrated in intranuclear foci (Figure 2A). The analysis of XYZ-axes projections and 3D rendering of OBs nuclear rims, pointed out their regular shape (Figure 2B,B’) and the presence of lamin B1 positive foci linked to nuclear lamina, that were mostly separated with occasional point of colocalization with lamin A/C (Figure 2A).

#### 2.2.2. SaOS2

**Figure 3:**
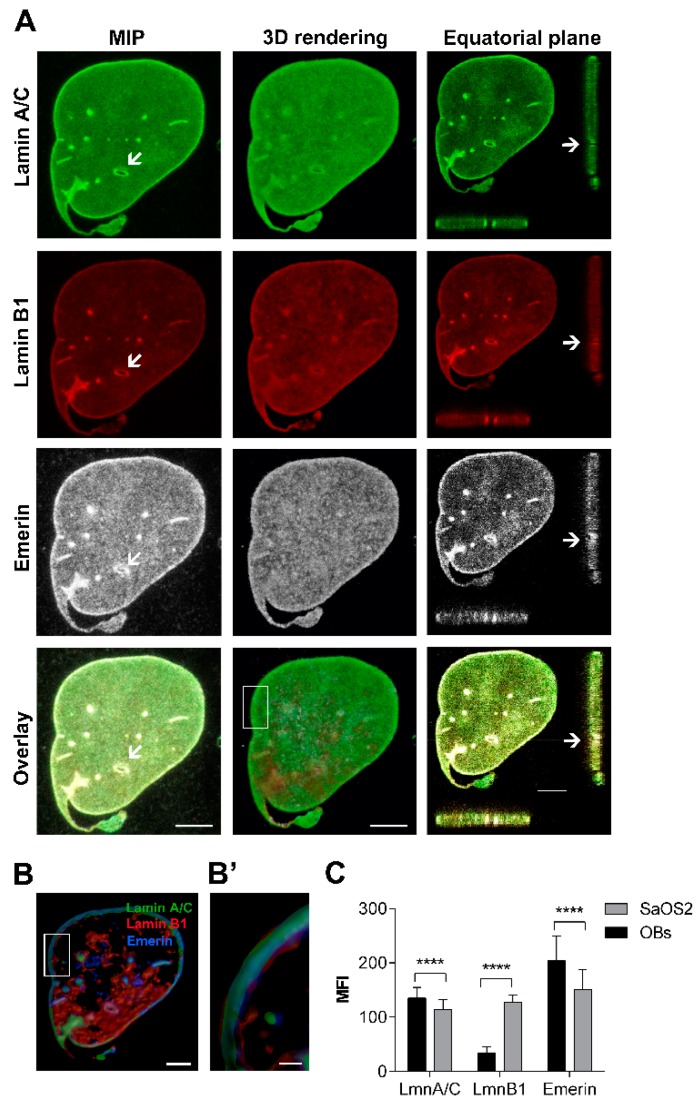
Behaviour of lamins and emerin, and nuclear morphology in SaOS2 cell line. (**A**) Maximum intensity projection (MIP) of series of z-stacks images covering the entire nucleus (left column), 3D rendering in blend volume mode (central column, LAS X 3D software, Leica Microsystems, Wetzlar, Germany), and XZ- and YZ-axes projections performed in the central focal plane of the nucleus (right column) are shown. The main morphological nuclear alterations as foci, folds, blebs and channels (arrow) are found in SaOS2 cells. Bars: 5 µm. (**B**) 3D rendering in the focal central plans of the nuclear rim in surface volume mode (Imaris software). Bar: 4 µm. (**B’**) High magnification of the 3D rendering of the boxed area showing the differential expression of lamins (in green and red) and emerin (pseudocoloured in blue). Bars: 1 µm. (**C**) Mean Fluorescence Intensity (MFI) of lamins and emerin immunoexpression in SaOS2 cells, in comparison to normal OBs. ****: *p* < 0.001.

Confocal imaging of multiple stained SaOS2 cells showed a higher number of morphological changes regarding shape, size and fragmentations in about 88.94 ± 15.80% of NEs (Figure 1C and Figure 3). Differently from OBs, SaOS2 cells presented numerous foci and channels (Figure 3A, arrow) labelled with both lamins and emerin antibodies (Figure 3B,B’).

#### 2.2.3. MG63

**Figure 4:**
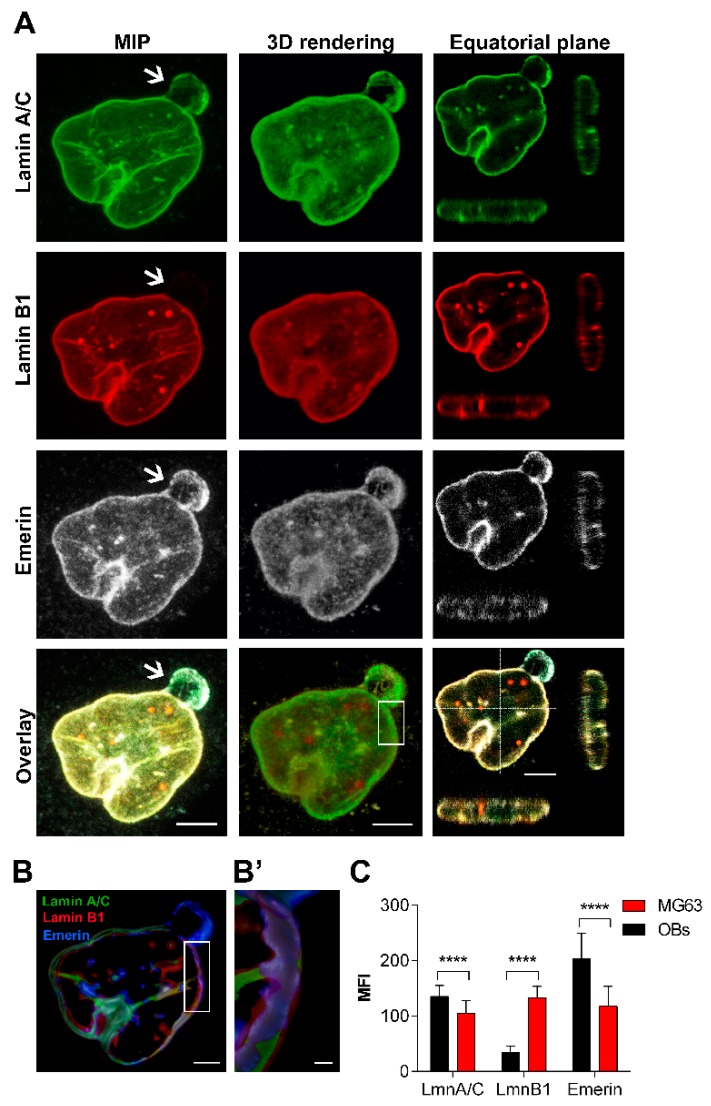
Behaviour of lamins and emerin, and nuclear morphology in MG63 cell line. (**A**) Maximum intensity projection (MIP) of series of z-stacks images covering the entire nucleus (left column), 3D rendering in blend volume mode (central column, LAS X 3D software, Leica Microsystems, Wetzlar, Germany), and XZ- and YZ-axes projections performed in the central focal plane of the nucleus (right column) are shown. Bars: 5 µm. (**B**) 3D rendering in the focal central plans of the nuclear rim in surface volume mode (Imaris software). Bar: 3 µm. (**B’**) High magnification of the 3D rendering of the boxed area showing the differential expression of lamins (in green and red) and emerin (pseudocoloured in blue). Bar: 1 µm. (**C**) Mean Fluorescence Intensity (MFI) of lamins and emerin immunoexpression in MG63 cells, in comparison to normal OBs. ****: *p* < 0.001.

Immunofluorescence analysis of MG63 cells performed by confocal microscopy detected a higher number of nucleoskeletal dysmorphisms in 84.30 ± 13.41% of cells than normal OBs (Figure 1A,B), mainly represented by blebs and honeycombs composed by a lamin A/C and emerin meshwork but lacking lamin B1 (Figure 4A arrow). Several foci were detected in the mid-plane section of MG63 nuclear rim, that were particularly enriched in lamin B1 and colocalizing with lamin A/C (Figure 4A). Overall, the intensity profiles of the lamins and emerin were brilliant (Figure 4B,B’), and the frequency of their mean value distribution were significantly different, being lamin A/C and emerin less expressed in MG63 than OBs, while lamin B1 mean fluorescence intensity was higher in the osteosarcoma cell line, in comparison to normal OBs (Figure 4C).

#### 2.2.4. HOS

**Figure 5:**
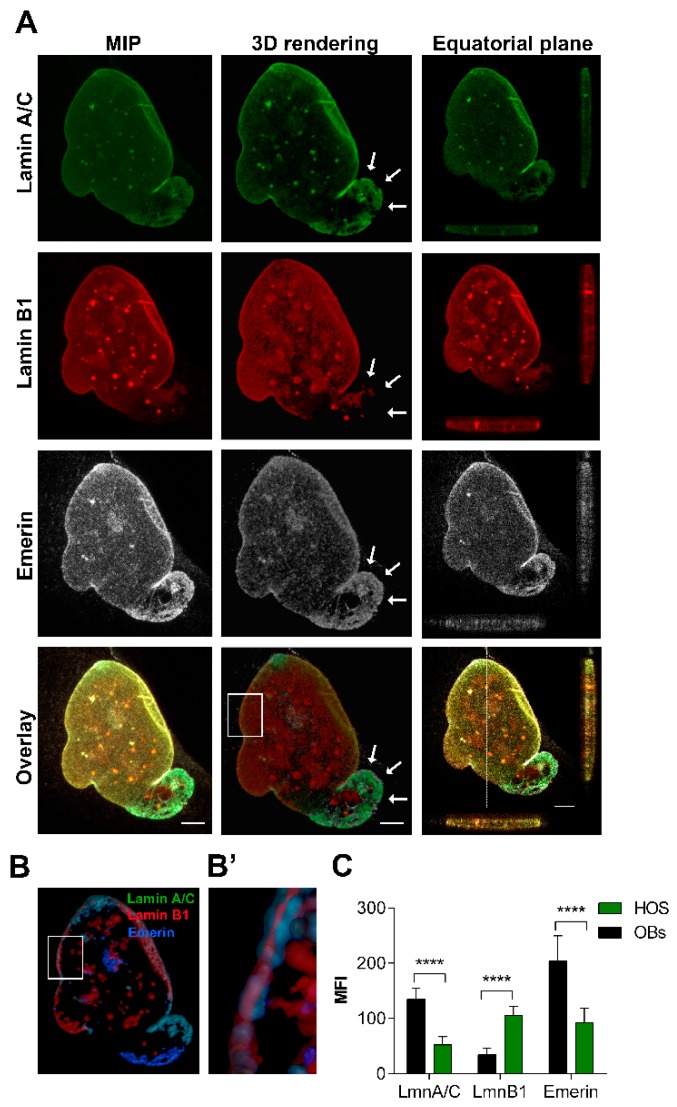
Behaviour of lamins and emerin, and nuclear morphology in HOS cell line. (**A**) Maximum intensity projection (MIP) of series of z-stacks images covering the entire nucleus (left column), 3D rendering in blend volume mode (central column, LAS X 3D software, Leica Microsystems, Wetzlar, Germany), and XZ- and YZ-axes projections performed in the central focal plane of the nucleus (right column) are shown. Bars: 5 µm. Lamin A/C (red) decreased and emerin (white) was mildly polymerized and interdispersed around the nuclear rim and in the cytoplasm. (**B**) 3D rendering in the focal central plans of the nuclear rim in surface volume mode (Imaris software). Bar: 3 µm. (**B’**) High magnification of the 3D rendering of the boxed area showing the differential expression of lamins (in *green* and *red*) and emerin (pseudocoloured in blue). Bar: 1 µm. (**C**) Mean Fluorescence Intensity (MFI) of lamins and emerin immunoexpression in HOS cells, in comparison to normal OBs. ****: *p* < 0.001.

A great nuclear size variability, a reduction in lamin A/C and, to a lesser extent, of emerin immunoexpression, were detected in HOS cells (Figure 5A,B). A number of about 77.01 ± 21.15% of cells showed lobulated NEs and/or honeycombs stained by lamin A/C and emerin, but devoid of lamin B1 (Figure 1 and Figure 5A, arrows). In addition, emerin was interspersed around the nuclear rim and in the cytoplasm. Numerous foci labelled by lamin B1, some of them partially stained by lamin A/C and emerin, were also detected (Figure 5A,B’). Significant differences of both emerin and lamins were detected in the distribution frequency of the fluorescence intensity values between HOS cells and normal OBs (Figure 5C).

#### 2.2.5. 143B

**Figure 6:**
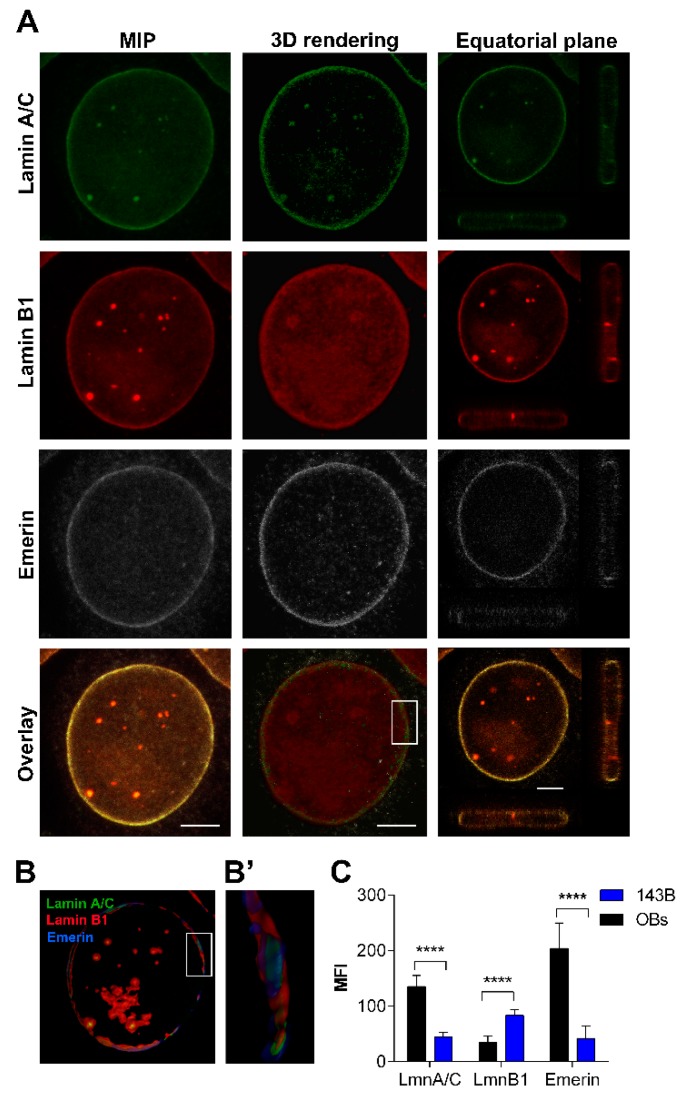
Behaviour of lamins and emerin, and nuclear morphology in 143B cell line. (**A**) Maximum intensity projection (MIP) of series of z-stacks images covering the entire nucleus (left column), 3D rendering in blend volume mode (central column, LAS X 3D software, Leica Microsystems, Wetzlar, Germany), and XZ- and YZ-axes projections performed in the central focal plane of the nucleus (right column) are shown. Bars: 5 µm. Lamin A/C (red) decreased and emerin (white) was mildly polymerized and interdispersed around the nuclear rim and in the cytoplasm. (**B**) 3D rendering in the focal central plans of the nuclear rim in surface volume mode (Imaris software). Bar: 3 µm. (**B’**) High magnification of the 3D rendering of the boxed area showing the differential expression of lamins (in green and red) and emerin (pseudocoloured in blue). Bar: 1 μm. (**C**) Mean Fluorescence Intensity (MFI) of lamins and emerin immunoexpression in 143B cells, in comparison to normal OBs. ****: *p* < 0.001.

Nucleoskeletal dysmorphisms were detected in about 68.66 ± 23.94% of 143B, a lesser value compared to HOS, MG63 and SaOS2 cell lines (Figure 1A). These features were mainly represented by folded NEs (Figure 1B) and, to a lesser extent, by lobulations. A very low positivity of lamin A/C and emerin was reported in 143B cells, whereas lamin B1 was higher in 143B cells in comparison to normal OBs (Figure 6A,C). Further, emerin was also interspersed in the cytoplasm of 143B cells. Intranuclear foci, enriched by lamin B1 and mildly by lamin A/C, were detected (Figure 6A). The comparison of mean fluorescence intensity values showed significant differences between 143B cell and normal OBs (Figure 6C).

**Figure 7:**
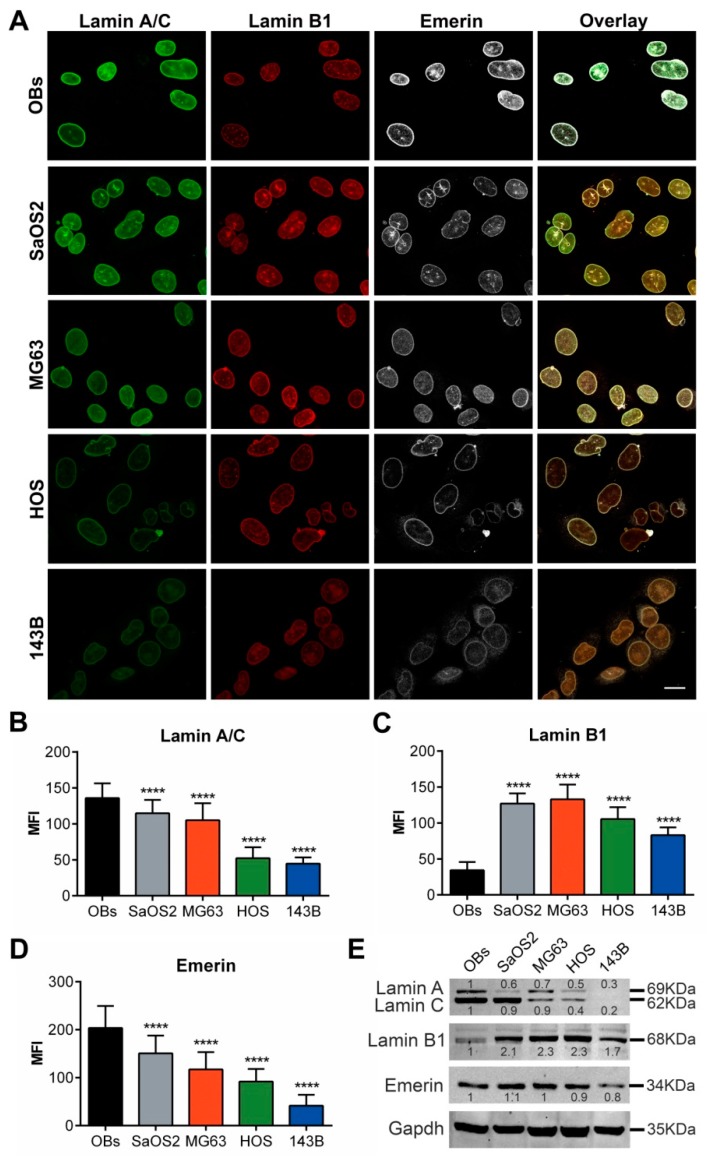
Main features of A/C and B1 lamins and emerin stained nuclei in osteoblasts and osteosarcoma cell lines. (**A**) Representative confocal images showing the differential expression of A/C and B1 lamins and emerin associated to their nucleoskeleton dysmorphisms. Bar: 20 µm. Mean Fluorescence Intensity (MFI) of lamin A/C (**B**), lamin B1 (**C**) and emerin (**D**) in OBs and osteosarcoma cell lines. **** *p* < 0.001. (**E**) Western blot analysis of lamin A, lamin C, lamin B1 and emerin in OBs and osteosarcoma cell lines, normalized versus the housekeeping Gapdh. The quantification of the bands for each protein is expressed as modulation with respect to normal OBs.

A global assessment of each nuclear protein in osteosarcoma cell lines revealed an inverse correlation between lamin A/C and emerin immunoexpression and the tumor cell aggressiveness, with a gradual reduction of these NE proteins starting from normal osteoblasts to high aggressive 143B cells (Figure 7A–D). As regarding lamin B1, all osteosarcoma cell lines showed higher levels than normal osteoblasts. Among tumor cells, 143B cells showed the lowest amount of lamin B1 (Figure 7C). Fluorescence intensity distributions and statistical analyses for each protein are reported in Supplementary Figure S1A–C. Western blot analysis confirmed the modulation of nuclear proteins among osteosarcoma cells and osteoblasts described by confocal microscopy assessment (Figure 7E) and revealed a different ratio of lamin A and lamin C protein amount in the different cells, although both isoforms decrease in relationship to cell line aggressiveness.

Comparing morphological nucleoskeletal changes and the immunoexpression data among osteoblasts and osteosarcoma cell lines (Figure 1 and Figure 7), we found a clear association between higher lamin B1 expression levels and nuclear dysmorphisms in osteoblastic cell lines; particularly SaOS2 cells, showing the higher variability of alterations, i.e., donut-shaped or fragmented nuclei, or NEs with blebs, septa and folds, exhibited high values of lamin B1 immunoexpression (Figure 7A,C). The nuclear lobulations and honeycombs, in all cell types, were enriched in lamin A/C and emerin but lacked lamin B1, whereas foci were strongly positive for both lamins and emerin. The differential expressions of these NE proteins among osteosarcoma cell lines were confirmed by RT-PCR (Supplementary Figure S2). Notably, no expression of progerin (mutated Δ50-A-type lamin) was found in osteosarcoma cells (data not shown).

### 2.3. Functional Effects Mediated by Lamin A/C Modulation on Cell Aggressiveness

**Figure 8:**
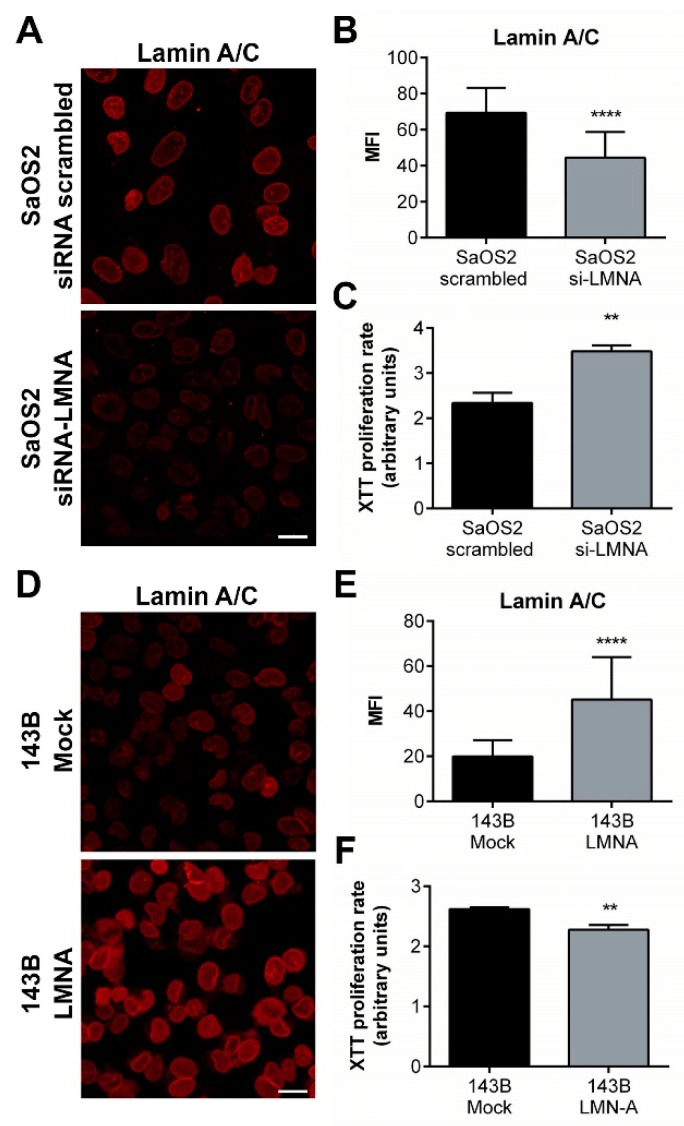
Modulation of LMNA gene expression in osteosarcoma cells. (**A**) Representative confocal images showing the differential expression of lamin A/C in SaOS2 cells treated with a scrambled siRNA (SaOS2 siRNA scrambled) and with a siRNA against LMNA gene (SaOS2 siRNA-LMNA). Bar: 30 μm. (**B**) Mean Fluorescence Intensity (MFI) of lamin A/C in siRNA scrambled vs. siRNA-LMNA SaOS2 cells. **** *p* < 0.001. (**C**) XTT proliferation assay performed on SaOS2 cells treated with siRNA scrambled and siRNA-LMNA for 72 h. ** *p* < 0.01. (**D**) Representative confocal images showing the differential expression of lamin A/C in 143B cells transfected with an empty vector (143B Mock) and with a plasmid conveying LMNA gene (143B LMNA). Bar: 30 μm. (**E**) Mean Fluorescence Intensity (MFI) of lamin A/C in Mock vs. LMNA 143B cells. **** *p* < 0.001. (**F**) XTT proliferation assay performed on 143B cells transfected with empty vector and LMNA-plasmid. ** *p* < 0.01.

In order to attribute a functional meaning to the differential content of A-type lamins in osteosarcoma cells with different aggressiveness, the expression of lamin A/C was downregulated in SaOS2 cells, that exhibited high levels of endogenous lamin A/C, as well as over-expressed in 143B cells, that showed a very low content of endogenous lamin A/C. Our findings demonstrate that the reduction of lamin A/C expression in SaOS2 cells leads to an increase in proliferation, while an opposite effect was obtained by an increase of lamin A/C expression in 143B cells, whose proliferation was found to be reduced (Figure 8).

### 2.4. Prognostic Relevance of Lamins and Emerin Expression on a TMA

To provide a prognostic significance of lamins and emerin expression, we assessed NE protein expression of 50 osteosarcoma samples on a tissue microarray (TMA) slide by immunofluorescence staining. The lamin A/C, lamin B1 and emerin fluorescence intensities, assessed by confocal microscope analysis, were evaluated and quantified and the results were compared to lamins and emerin immunoexpression in a normal bone sample.

**Figure 9:**
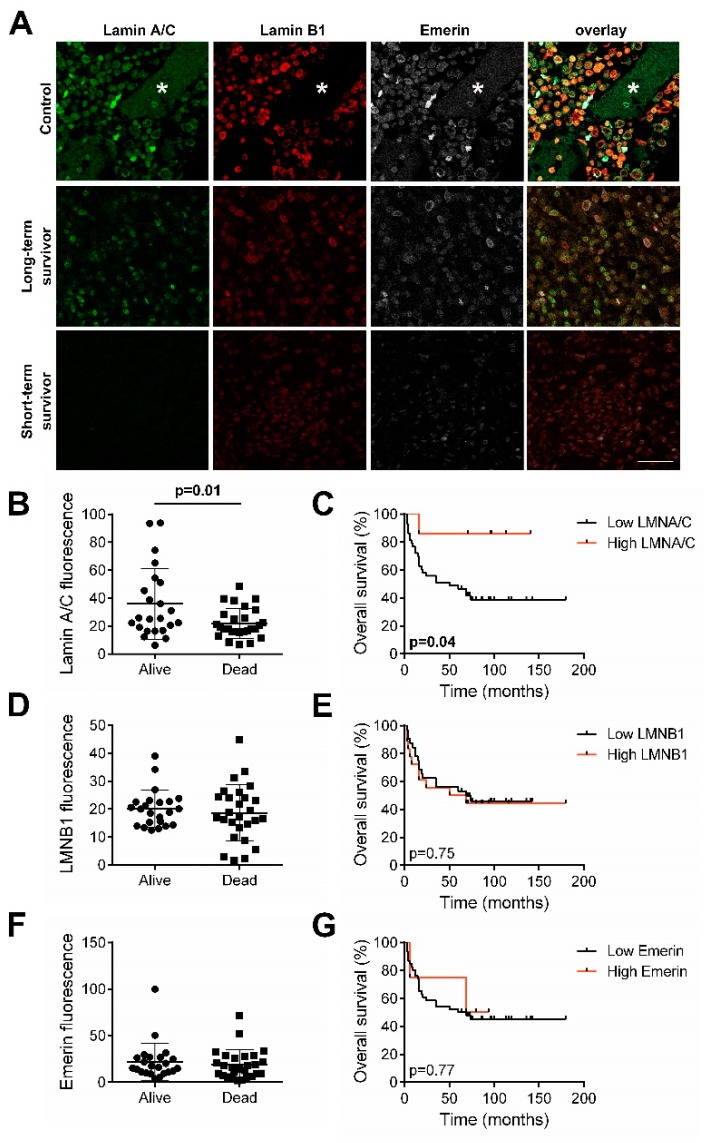
(**A**) Confocal imaging of lamin A/C (green), lamin B1 (red) and emerin (white) in bone tissue from controls and osteosarcoma patients with different overall survival: long-term survivor (alive) patient and short-term survivor (dead) patient. A bone trabeculae was visible in healthy tissue (asterisk). Bar: 30 µm. (**B**) Intensity distribution of lamin A/C fluorescence in long-term versus short-term osteosarcoma patients from TMA analysis. (**C**) Kaplan–Meier curves showing overall survival for patients with low- and high- lamin A/C staining. (**D**) Intensity distribution of lamin B1 fluorescence in long-term versus short-term osteosarcoma patients from TMA analysis. (**E**) Kaplan–Meier curves showing overall survival for patients with low- and high- lamin B1 staining. (**F**) Intensity distribution of emerin fluorescence in long-term versus short-term osteosarcoma patients from TMA analysis. (**G**) Kaplan–Meier curves showing overall survival for patients with low- and high-emerin staining.

Samples representing long-term and short-term survival osteosarcoma patients were shown in Figure 9A. Long-term survival patient showed high amount of lamin A/C, lamin B1 and emerin expression (Figure 9A). In contrast, a sample obtained from short-term survival osteosarcoma patient expressed high levels of lamin B1 and no detectable A/C-type lamin and emerin (Figure 9A).

The immunoexpression of lamin A/C evaluated by confocal image analysis was correlated with patients’ overall survival (OS), finding a statistically significant positive correlation between long-term survival patients and lamin A/C expression (Figure 9B). The Kaplan–Meier curves (Figure 9C) confirmed a better prognosis for patients showing higher lamin A/C staining (fluorescence intensities from value 45–93) than patients with low lamin A/C staining (fluorescence intensities from value 6–44). As regarding lamin B1 and emerin, no significant correlation was found between their immunoexpression and patients’ overall survival (Figure 9D–G).

### 2.5. Linking Nucleoskeleton to Cytoskeleton: The Altered Nuclear Shuttling of MKL1 Follows the Lamin A/C-Emerin Behavior and the Polymerization Status of Actin

Since nuclear translocation of endogenous MKL1, in response to serum stimulation, is abrogated in *Lmna^−/−^* mouse embryonic fibroblasts [40], the subcellular localization of MKL1 was assessed in osteosarcoma cell lines with different lamin A/C expression. In line with these prior observations, we found that in 143B cells, the reduced lamin A/C expression correlates with a severely altered nucleo-cytoplasmic MKL1 distribution, with a substantial amount of MKL1 still in the cytoplasm.

**Figure 10:**
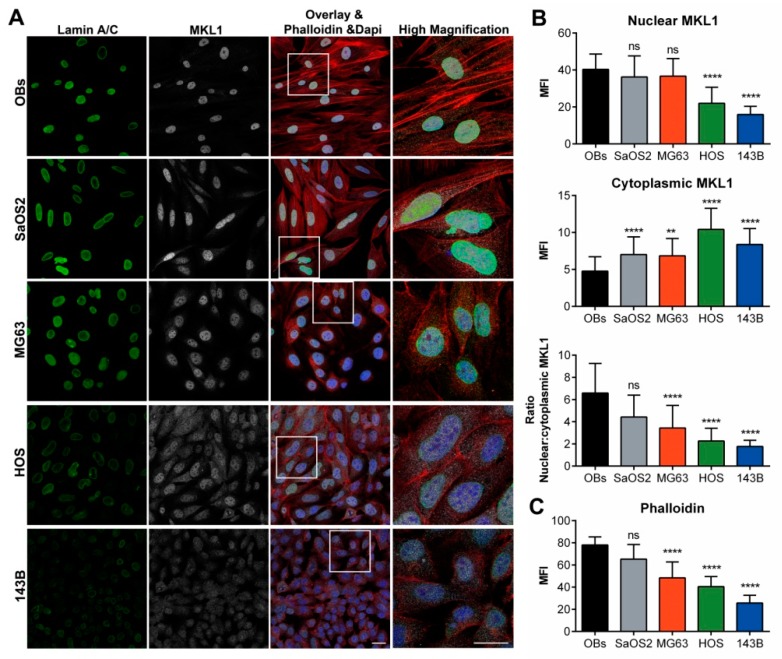
Altered nucleo-cytoplasmic MKL1 shuttling and different polymerization status of actin were differentially observed in osteosarcoma cell lines. (**A**) Subcellular distribution of MKL1 (white), actin (red) and lamin A/C (green) Nuclei were stained with Dapi. Bar: 30 µm. (**B**) Mean Fluorescence Intensity (MFI) of nuclear (upper panel), cytoplasmic (middle panel) and the ratio nuclear-cytoplasmic (lower panel) MKL1 in OBs and osteosarcoma cell lines. (**C**) Mean Fluorescence Intensity (MFI) of phalloidin in OBs and osteosarcoma cell lines. ** *p* < 0.01, **** *p* < 0.001; ns: not significant.

In accordance to the decreasing lamin A/C expression starting from normal OBs to 143B cells (Figure 7), we observed a parallel gradual MKL1 subcellular re-distribution, towards a prevalent nuclear localization found in control OBs in canonical serum condition (Figure 10A,B). Nuclear MKL1 fluorescence intensity distributions among the different cell lines and the relative statistical analyses are reported in Supplementary Figure S1D.

In parallel, the polymerization status of actin followed the different lamin A/C expression among normal OBs and osteosarcoma cell lines, in particular in HOS and 143B cells in which the severely altered nucleo-cytoplasmic MKL1 distribution was accompanied by a reduced actin polymerization (Figure 10A,C). Fluorescence intensity distributions and statistical analyses for phalloidin are reported in Supplementary Figure S1E. These findings suggest a close correlation between lamin A/C expression and functional NE features and, consequently, of G-actin transport in osteosarcoma cells.

### 2.6. Functional Effects of Differential Expression of A-Type Lamin in Osteosarcoma Cells

**Figure 11:**
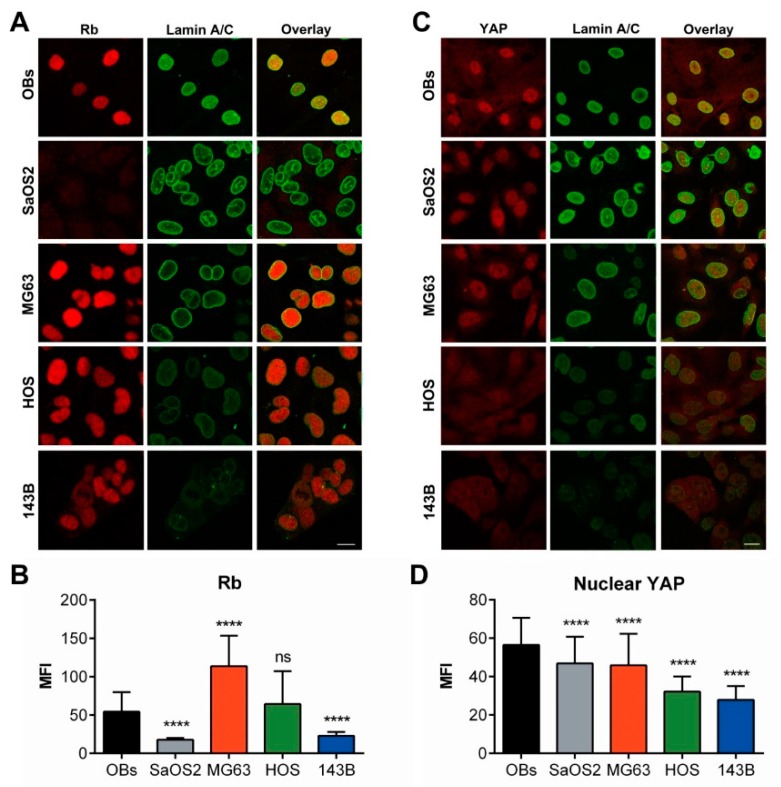
Altered expression of pRb and YAP proteins correlates with lamin A/C expression. (**A**) Confocal imaging of Rb (*red*) and lamin A/C (green) in osteoblasts (OBs) and osteosarcoma cell lines. Rb immunolabeling was reduced in HOS and particularly in 143B cells respect to MG63 cells, whereas SaOS2 cells resulted negative. (**B**) Mean Fluorescence Intensity (MFI) of Rb in OBs and osteosarcoma cell lines. (**C**) YAP immunoreaction was observed in the nuclei of OBs, SaSO2 and MG63 cells whereas it was significantly reduced in higher aggressive cells (HOS and 143B). (**D**) Mean Fluorescence Intensity (MFI) of nuclear YAP in OBs and osteosarcoma cell lines. Bars: 20 μm. **** *p* < 0.001; ns: not significant.

In order to functionally characterize the alteration of nuclear envelope proteins in osteosarcoma cells, we addressed the expression of protein Retinoblastoma (pRb) in relationship to A-type lamin content, starting from the observation that mouse fibroblasts deficient for A-type lamin showed reduced levels of pRb [43]. Indeed, we found a gradual deregulation of pRb consistent with the reduction of A-type lamin observed in the high aggressive osteosarcoma cell lines in comparison to low aggressive tumor cells and normal OBs (Figure 11A), also confirmed by fluorescence quantification analysis (Figure 11B).

pRb fluorescence intensity distributions and the relative statistical analyses of multiple comparisons among assessed cell lines are reported in Supplementary Figure S1F. Likewise, the nuclear localization of the Yes-Associated Protein (YAP), known to be related to A-type lamin expression [36], was reduced in high aggressive cells in comparison to low aggressive osteosarcoma cells or normal osteoblasts, following the same reduction pattern of A-type lamin (Figure 11C), as confirmed by fluorescence quantification analysis (Figure 11D). Supplementary Figure S1G shows the fluorescence intensity distribution and the statistical analysis of nuclear YAP.

## 3. Discussion

In this study, we deepened the behavior of pivotal components of the nuclear envelope, type A/C and B1 lamins and emerin, in different osteosarcoma cell lines with different malignant aggressiveness, in comparison to human normal osteoblasts. Osteosarcoma cells are characterized by extreme aneuploidy and chromosomal instability [45], to such an extent that osteosarcomas are generally used as a prototype to study the chromosomal instability due to their complex karyotypes including translocations and a high number of chromosomal amplifications and deletions, suggesting that genomic instability is a key component of the osteosarcoma pathogenesis. Unlike many sarcomas, which are characterized by specific chromosome translocations, individual osteosarcoma cells are characterized by complex genomic rearrangements involving any chromosome [46]. Exposure to ionizing radiation, for example during radiotherapy of other cancers, is a high-risk factor for the inset of osteosarcoma at the irradiated site, further suggesting that DNA breakage and genomic instability contribute to the development of this specific type of tumor [47]. Despite the well-known notion about nuclear chromosome alterations in osteosarcoma, this is the first evidence of nuclear phenotypes resembling specific laminopathic nuclear alterations in osteosarcoma cells. The involvement of NE proteins in tumorigenesis process is still controversial. Patients with laminopathies, other than progeria, with a relatively long life expectancy do not develop cancer in more cases than expected. The work by Therizols et al. [48] could explain this observed phenomenon: mice lacking ZMPSTE24, the protease that processes prelamin A to lamin A, develop multiple abnormalities and progeroid features, due to the prelamin accumulation and the reduced effective A-type lamin availability. In these mice, p53 signalling appears to be enhanced, leading to a p53-mediated entry into a senescence program that seems a mechanism used by cells to suppress the development of cancer [49]. At the same time, DNA mutation accumulation, especially those that inactivate either the p53 or Rb pathways, occurred in aging process caused by A-type lamin signalling alteration, as well as in cancer progression, suggesting that organisms likely respond to DNA damages either with elevated cancer incidence or with accelerated aging [50].

Starting from this notion, many types of cancer present with lamin alterations. The main changes in lamins expression are aberrant localization and reduction in expression of A-type lamins that frequently correlate with cancer subtypes and cancer aggressiveness, proliferative capacity and differentiation state.

Although lamins are altered in many type of tumors, the counterpart condition, that is cancer development in laminopathic patients, is a rare event, but the only tumor described in association with progeria syndrome patients (the sample size is quite small) is an early onset osteosarcoma [27,29]. All together, these observations from the literature prompted us to deeper investigate the expression and function of NE proteins in osteosarcoma cells. The nuclear dysmorphisms observed in osteosarcoma cells closely resemble peculiar laminopathic nuclear phenotypes. To the best of our knowledge, this is the first depth morphological characterization of NE architecture of tumoral cells showing donut nuclei, honeycombs folds, and blebs. Indeed, the percentage of dysmorphic nuclei in all the tested osteosarcoma cells resulted higher than normal osteoblasts. Among assessed osteosarcoma lines, the low aggressive SaOS2 cells showed the highest content of nuclear morphology alterations. In our opinion, this could be explained by considering the proliferation rate of SaOS2 cells, which is higher than normal osteoblasts but lower than aggressive osteosarcoma cells. In view of this, we hypothesize that the high proliferation rate of the high aggressive 143B cells could prevent the accumulation of dysmorphic nuclei, although the dramatic modulation of the A-type lamin expression in these cells. Indeed, a correlation between nuclear dysmorphism accumulation and cellular proliferation was found in several tumor cells [51,52,53], suggesting that highly proliferating cells may have more nuclear abnormalities than slowly proliferating cells. However, in our conditions, we observed that SaOS2 cells accumulates nuclear abnormalities and, at the same time, they are low proliferative cells. In support of this, we found in the literature some papers in which nuclear abnormalities are found in low proliferative cells: a high amount of nuclear dysmorphisms have been described in aged, low proliferative iPSC cells and in senescent cells [8,54]. In addition to increased nucleoskeletal dysmorphisms, osteosarcoma cell lines have significant alteration of A-type and B-type lamins, as well as of emerin expression in comparison to normal osteoblasts. In particular, the content of A-type lamin and emerin is reduced in osteosarcoma cells in comparison to normal osteoblasts and this reduction correlate with tumor aggressiveness. More in details, western blot analysis revealed a different content for lamin A and lamin C in each cell lines, being lamin C the more expressed A-type isoform and better related to osteosarcoma aggressiveness. On the contrary, B-type lamin is always overexpressed in osteosarcoma cells in comparison to normal osteoblasts. These data suggest the feasibility to use NE protein expression as potential prognostic and/or diagnostic biomarkers for osteosarcoma, as already described for other cancers [52].

To demonstrate the functional effects of lamin A/C in osteosarcoma features, we reversed the basal lamin A/C expression in low aggressive SaOS2 cells, obtaining a worsening of the phenotype, and in high aggressive 143B cells, observing an amelioration of the tumor features. These results strongly support our working hypothesis of the crucial roles exerted by nuclear lamins in the onset and/or progression of osteosarcoma.

Starting from the observation that lamin A/C was strongly correlated to the aggressiveness of osteosarcoma cell lines, the expression of this nucleoskeleton protein in histological samples has provided a prognostic significance for lamin A/C expression, pointing out that the high lamin A/C staining can be considered as a “good prognosis” predictor in osteosarcoma patients. These data are in accordance with other works in the literature in which the reduction of lamin A/C expression is correlated to an increased aggressiveness of the tumor [55,56,57].

As regarding the functional effects dependent on NE protein deregulation in osteosarcoma cells, we described an altered cytoplasm-nuclear shuttling of MKL1 in association with reduced A-type lamin and emerin. In accordance with the work of Willer and coauthors [41], our results clearly showed a reduced nuclear shuttling of MKL1 in those osteosarcoma cells with reduced expression of both A-type lamin and emerin. Starting from the aforementioned notion that the SRF−MKL1 activation mediated by A-type lamin and emerin activity is essential in cells resident on a stiff matrix, the significance of this alteration is exacerbated in osteosarcoma, a tumor arising in the bone, the stiffest tissue of our body.

Regarding pRb, in our conditions, high aggressive osteosarcoma cells, with reduced A-type lamin and emerin expression, showed reduced amount of nuclear pRb, confirming the aforementioned association between lamins and the Retinoblastoma protein. Moreover, these findings suggest a potential mechanism by which aggressive osteosarcoma cells inactivate the oncosuppressor pRb, thereby promoting their proliferation.

Swift and coauthors have demonstrated the A-type lamin-dependent activation of the Yes-Associated Protein (YAP) in cells resident on a stiff matrix [36], therefore we addressed the nuclear content of YAP in osteosarcoma cells showing modulated NE protein expression. Indeed, also in this case, we confirm a correlation between A-type lamin/emerin expression and the YAP nuclear localization. YAP being, together with MKL1, a mechanosensitive regulator of gene transcription [58], all together these results strongly suggest a great component of mechanical cues in the onset and progression of osteosarcoma, mechanism that requires deeper investigation.

## 4. Materials and Methods

### 4.1. Cell Lines and Culture

Human osteosarcoma cell line SaOS2 (HTL01001), MG-63 (HTL99003) and HOS (HTL04003) were purchased from Banca Biologica and Cell Factory (IRCCS Azienda Ospedaliera Universitaria San Martino-IST, Genova, Italy), while osteosarcoma cell line 143B (CRL-8303) was purchased from American Type Culture Collection (Manassas, VA, USA). Primary human femoral osteoblasts HOB (4610) was purchased from ScienCell™ (Carlsbad, CA, USA). All cell types were grown in Dulbecco’s modified Eagle’s medium (DMEM) (Euroclone, Milan, Italy) supplemented with 10% Fetal Bovine Serum (FBS), 100 units/mL penicillin/streptomycin (Euroclone) and maintained at 37 °C in 5% CO_2_.

### 4.2. Immunofluorescence

5 × 10^3^ cells were seeded into plastic-chambered glass microscope slides (BD Falcon), fixed with 4% paraformaldehyde in PBS for 10 min followed by PBS/Triton 0.1% for 5 min. The following primary antibodies were used according to manufacturer instructions: mouse anti-lamin A/C and goat anti-lamin A/C purchased from Santa Cruz Biotechnology (Temecula, CA, USA), mouse anti-emerin (Leica, Mannheim, Germany), rabbit anti-lamin B1 (Abcam, Cambridge, UK), rabbit anti-MKL1 (Sigma-Aldrich, St. Louis, MO, USA), mouse anti-pRb (Cell Signaling Technology, Danvers, MA, USA) and rabbit anti-YAP1 (Santa Cruz Biotechnology, Dallas, TX, USA) antibodies. Secondary antibodies conjugated with Alexa Fluor-488, -555 and -647 dyes (Life technologies) were used diluted in 1% PBS/BSA for 1 h, RT. F-actin and nuclear acids were stained using Phalloidin-TRITC and Dapi (Life technologies, Carlsbad, CA, USA), respectively. Slides were mounted with PBS/glycerol 1:1. Negative controls were performed in each labeling using 1% PBS/BSA without the primary antibody, to verify specific staining. Bone and osteosarcoma biopsies were used to perform immunofluorescence on 2-μm thick sections obtained from formalin-fixed tissue embedded in paraffin. Antigen retrieval was performed with ethylenediaminetetraacetic acid (EDTA) (pH 9) (Dako, Glostrup, Denmark). Then sections were incubated with lamins and emerin antibodies as described above.

### 4.3. Confocal Microscopy and Image Analysis

Confocal microscopy was performed on a Leica TCS-SP8X laser-scanning confocal microscope (Leica Microsystems, Mannheim, Germany) equipped with tunable white light laser (WLL) source, 405 nm diode laser, 3 Internal Spectral Detector Channels (PMT) and 2 Internal Spectral Detector Channels (HyD) GaAsP. Sequential confocal images were acquired using a HC PL APO 60x oil-immersion objective (1.40 numerical aperture, NA, Leica Microsystems, Mannheim, Germany) with a 1024 × 1024 format, scan speed 400 Hz, and z-step size of 0.25 µm. Fluorochromes unmixing was performed by acquisition of automated-sequential collection of multi-channel images, in order to reduce spectral crosstalk between channels. Lasers’ power, beam splitters, filter settings, pinhole diameters and scan mode were the same for all examined samples of each staining. To improve contrast and resolution of confocal raw images, deconvolution analysis (Deconvolution software, Leica Microsystems, Wetzlar, Germany) was applied to Z stacks, then deconvolved images were imported into LAS X 3D (Leica Microsystems) or IMARIS (Bitplane, Zurich, Switzerland, CH) software to obtain their surface 3D reconstruction to a better visualization of nucleoskeletal architectures. Maximum intensity projection (MIP) of z-series and Z-stack profiles in the individual nuclei of image series (across the entire z-stack), based on region of interest (ROI = single nucleus), were obtained by LAS X (Leica Microsystems) software. The quantitative analysis of immunostained cell samples was performed on single confocal images acquired in the focal central plane of cells, in which the nuclear rim is perfectly visible. Nuclear abnormalities such as folds, blebs, honeycombs, fragmented and donut nuclei were counted in each cell line (# of assessed nuclei: 156 for OBs, 251 for SaOS2, 253 for MG63, 170 for HOS and 181 for 143B cells), and the mean fluorescence intensity of lamin A/C, lamin B1 and emerin in the nuclear rim was manually measured for each nucleus, then calculated using LAS X software. A number >130 nuclei from ten single section images (40× magnification, using a HC PL APO oil-immersion objective, 1.40 NA) was randomly selected and analyzed for each cell sample. The MKL1 mean fluorescence intensity was measured by ImageJ software from cytometric measurements relative to nuclear and cytoplasmic compartments, in 5 single section images (40× magnification) randomly selected and analyzed for each cell sample. Tables of images were processed using Adobe Photoshop CS4 software (Adobe Systems Inc., San Jose, CA, USA).

### 4.4. Western Blotting

Cell pellets were collected, and total protein extraction was performed by homogenizing cells in Ripa lysis buffer (Cell Signalling Technology, Danvers, MA, USA) containing 1X protease and phosphatase inhibitors cocktail. The homogenates were then centrifuged at 13,000 rpm at 4 °C for 10 min and the resulting supernatant was taken as protein samples. Cell extracts were quantified using the BCA™ Protein Assay (Thermo Fisher, Foster City, CA, USA). Samples were then diluted in the sample buffer [200 mM Tris-HCl (pH 6.8), 20% β-mercaptoethanol, 4% sodium dodecyl sulphate, and bromophenol blue] and resolved in SDS-PAGE, then transferred and immobilized onto nitrocellulose membranes (Amersham). The membranes were blocked using 5% BSA for 30 min and incubated with the appropriate primary and secondary antibodies. Signal intensity was measured with a ChemiDoc Imaging System (BioRad, Hercules, CA, USA). Primary antibodies: mouse anti-lamin A/C purchased from Santa Cruz Biotechnology (Temecula, CA, USA), rabbit anti-lamin B1 (Abcam, Cambridge, UK), mouse anti-emerin (Leica, Mannheim, Germany), rabbit anti-Gapdh were supplied by Sigma-Aldrich (Milan, Italy). Densitometric assessment of the western blot bands was performed by ImageJ quantification. The content of each protein for each sample was normalized versus the relative Gapdh value and expressed as a modulation with respect of normal osteoblasts, except for Lamin C, that was expressed as a modulation of Lamin A of normal osteoblasts. The whole blots showing all the bands with all molecular weight markers on the Western blot are shown in Supplementary Figure S3.

### 4.5. RNA Interference Knockdown and Plasmid Over-Expression

IBONI^®^siRNA duplexes specific for human LMNA (iBONI siRNA LMNA) gene were purchased from Riboxx, and scrambled duplexes were used as control (iBONI siRNA scrambled). An amount of 5 × 10^4^ SaOS2 cells was plated in 12-well plates. At approximately 50% confluence, cells were transfected with the annealed siRNA-LMNA and siRNA-scrambled (siRNA final concentration 10 nM) using INTERFERin^®^ (Polyplus Transfection, New York, NY, USA). Cells were treated with siRNA for 48 h, then cells were fixed and assessed for lamin A/C content by immunofluorescence.

For lamin A/C over-expression in 143B cells, we purchased the pBabe-Puro-GFP-LMNA from Addgene, while an empty vector (pBabe-Puro-GFP) was used as control. An amount of 5 × 10^5^ 143B cells was plated in a 10 cm dish and trasfected with 3 μg of plasmid DNA by Lipofectamin 2000 (Invitrogen), according to the manufacturer’s instructions. Transfected cells were treated with puromicin (1 μL/mL) for 2 weeks, then cells were fixed and assessed for lamin A/C content by immunofluorescence.

### 4.6. XTT Proliferation Assay

For proliferation assay, cells were seeded at a density of 500 cells/well in 96-well plates and maintained for 72 h. Cell proliferation was analyzed using the XTT assay kit (ROCHE, Mannheim, Germany) according to the manufacturer’s instructions. Briefly, 50 μL of the activated XTT solution was added to each well and incubated for 4 h. Next, the absorbance of samples was measured with a spectrophotometer (ELISA reader) at a wavelength of 450 nm wavelength and a reference wavelength of 650 nm.

### 4.7. Osteosarcoma Patient’s Samples on a Tissue MicroArray (TMA)

Human samples used in this work were commercially available, therefore we did not need ethical approval or informed consent. A panel of 60 osteosarcoma specimens was analyzed using tissue microarray (TMA) slides CV2 human, osteosarcoma Super Biochips (Super Biochips, South Korea distributed by CliniSciences, Italy). Of them, 9 spots were not interpretable because of loss of tissue on the microarray slide or absence of tumor cells in the spot or loss of survival information, while 1 sample, obtained from an osteosarcoma lung metastasis, was censored from this study being the only one representative of this type of tumor and useless for any statistical analysis. Therefore, 50 tumor cores were interpretable for expression of lamin A/C and had associated survival information. The clinical-pathological data of the 50 osteosarcoma patient samples available for analysis are shown in Table 1. For each TMA core, the fluorescence intensity of *n* < 100 nuclei staining was scored by ImageJ software quantification. For those specimens that were uninterpretable, a score of not applicable (N/A) was assigned. The lamin A/C staining intensities, ranged from 6.3 to 93.7 values, were grouped as low (MFI values < 45) and high (MFI values > 45) staining in order to generate Kaplan-Meyer curve. The lamin B1 staining intensities, ranged from 1.6 to 44.8 values, were grouped as low (MFI values < 22) and high (MFI values > 22) staining in order to generate Kaplan–Meier curve. The emerin staining intensities, ranged from 2.1 to 100.1 values, were grouped as low (MFI values ≤ 50) and high (MFI values > 50) staining in order to generate Kaplan–Meier curve.

### 4.8. RNA Isolation, Reverse Transcription (RT) PCR Analysis

Total RNA was extracted from cultured cells using the standard Trizol procedure. Each RNA sample was quantified by NanoDrop 2000 (Thermo Fisher, Foster City, CA, USA). Two μg of RNA were reverse transcribed using the SuperScript™ II Reverse Transcriptase (Invitrogen- Thermo Fisher, Foster City, CA, USA) to generate cDNA. The polymerase chain reaction (PCR) has been carried out with Power SYBR Green dye chemistry (appliedbiosystems by Thermo Fisher Scientific) using the 7500 Fast Real-Time PCR System (Applied Biosystems, Foster City, CA, USA). Results have been normalized to human GAPDH levels using the 2^−ΔΔCt^ method. Primer pair sequences are shown in Table 2.

### 4.9. Statistical Analysis

Data were referred from at least three independent experiments on each cell line. All analyses were completed using GraphPad Prism 6.0 (San Diego, CA, USA). Difference in the percentage of dysmorphic nuclei among assessed cells were evaluated with one-way ANOVA analysis, Kruskal–Wallis test, and by Dunn’s multiple comparison test. Differences in the level of fluorescence intensity distributions were evaluated with Kruskal–Wallis, Mann–Whitney rank-sum test, and Dunn’s multiple comparison post-hoc test and *p* values < 0.05 were considered statistically significant. Survival curves were calculated using the Kaplan–Meier method and the Mantel-Cox log-rank test was employed for comparison.

## 5. Conclusions

In this work, we described for the first time a laminopathic phenotype in osteosarcoma tumor cells, showing, especially in SaOS2 cells, some of the peculiar nuclear dismorphisms so far observed in laminopathic pathological conditions. We also provided the evidence of an inverse correlation between the lamin A/C expression and the aggressiveness of four osteosarcoma cell lines, thereby identifying lamin A/C as a new prognostic factor for osteosarcoma patients. Due to the recent re-interpretation of nuclear envelope proteins as cellular mechanosensors, these findings suggest the involvement of mechanical cues in the onset and/or progression of osteosarcoma that deserves deeper investigations.

## Figures and Tables

**Table 1:** The clinical-pathological data of the osteosarcoma patients on tissue microarray (TMA).

Age	Cases/Total	%
≤18 years	25/50	50%
>18 years	25/50	50%
**Gender**		
Male	37/50	74%
Female	13/50	26%
**Type of Osteosarcoma**		
Conventional osteosarcoma:	29/50	58%
Osteoblastic type	20/29	69%
Chondroblastic type	8/29	28%
Fibroblastic type	1/29	3%
Telangiectatic osteosarcoma	1/50	2%
Extraskeletal osteosarcoma	2/50	4%
Metastatic osteosarcoma, chondroblastic type	1/50	2%
Synovial sarcoma	1/50	2%
Granulation tissue	1/50	2%
Radiation-induced sarcoma	1/50	2%
Osteosarcoma (not defined)	15/50	30%
**Site of Osteosarcoma**		
Bone, femur	23/50	46%
Bone, tibia	7/50	14%
Bone, fibula	2/50	4%
Bone, humerus	3/50	6%
Bone, pelvis	2/50	4%
Bone, ilium	2/50	4%
Bone, mandible	2/50	4%
Bone, tibia and fibula	2/50	4%
Bone, calcaneus	1/50	2%
Mediastinum	1/50	2%
Knee	1/50	2%
Hip	1/50	2%
Leg	1/50	2%
Soft tissue	2/50	4%
**Overall Survival**		
Alive (beyond 5 year-follow up)	29/50	48.30%
Dead (within 5 year-follow up)	29/50	48.30%

**Table 2:** Primer pairs used for RT-PCR.

Gene	Sequences	Product Size
Human LMNA	left: CTACACCAGCCAACCCAGAT	126 bp
	right: GGTCGAAGGACAGAGACTGC	
Human LMNB	left: AACGAGACCAGAAGGAAGCA	117 bp
	right: GGCATCATGTTGCTCTCTCA	
EMERIN	left: CAGTGCCTACCAGAGCATCA	232 bp
	right: AAAGACCAGGAAAAGCAGCA	
GAPDH	left: CGACCACTTTGTCAAGCTCA	228 bp
	right: AGGGGTCTACATGGCAACTG	

## Supplementary Materials

The following are available online at https://www.mdpi.com/2072-6694/12/2/443/s1, Figure S1: Fluorescence intensity frequency distributions of NE proteins in osteoblasts and osteosarcoma cell lines, Figure S2: RT-PCR analysis of NE protein expression in osteoblasts and osteosarcoma cell lines, Figure S3: Whole blots of western blot analysis.
