# Supplementary figures and images for: Nuclear Lamins and Emerin Are Differentially Expressed in Osteosarcoma Cells and Scale with Tumor Aggressiveness
Source: Cancers (Basel). 2020 Feb 13;12(2):443. doi: 10.3390/cancers12020443 (PMC7073215; doi:10.3390/cancers12020443)

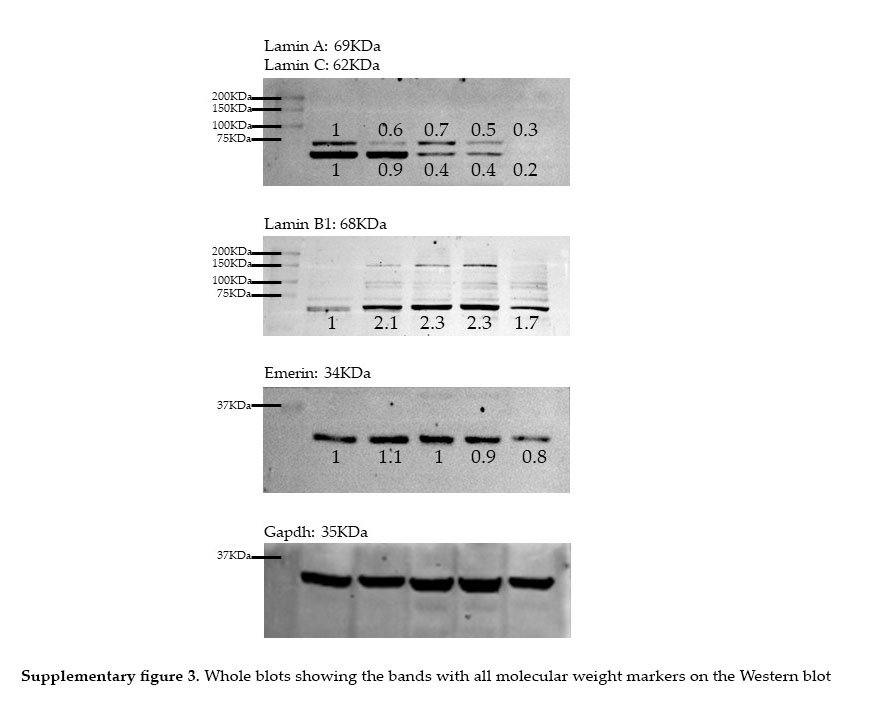

Supplement: Supplementary file 1 [file cancers-12-00443-s001.zip › Supplementary figure 3.jpg]
